# High-Throughput Screening Identifies Novel Antileishmanial Scaffolds and Reveals Cross-Species Activity of Structurally Diverse Compounds

**DOI:** 10.21203/rs.3.rs-10898149/v1

**Published:** 2026-09-30

**Authors:** Juan P. Gonzalez, Hannah N. Corman, Douglas A. Shoue, Mohanna Sadat Sajjadi Oskouei, Theodore O. Schroder, Chaniay O’Brien, Bruce J. Melancon, Mary Ann McDowell

**Affiliations:** University of Notre Dame; University of Notre Dame; University of Notre Dame; University of Notre Dame; University of Notre Dame; Kean University; University of Notre Dame; University of Notre Dame

**Keywords:** Leishmaniasis, drug discovery, high-throughput screening, drug optimization, structure-activity relationship

## Abstract

**Background:**

Leishmaniasis remains a neglected tropical disease with limited therapeutic options and a continued need for new and effective treatments. High-throughput phenotypic screening provides an approach for identifying chemically diverse compounds with antileishmanial activity that can serve as a starting point for drug development. This study further characterized compounds identified through a previous high-throughput phenotypic screen to prioritize promising antileishmanial chemical scaffolds and evaluate their potential for continued drug development.

**Methods:**

Compounds belonging to two enriched scaffolds, 1,4-diaryl-pyrazolo-pyridinone (1,4-DAPP) and pyrazolo-[5,1-c][1,2,4]triazine (PTZ), along with structurally diverse singleton compounds, were evaluated for antileishmanial activity across *Leishmania* parasite life stages by determining half-maximal inhibitory concentrations (IC_50_). Host-cell cytotoxicity was evaluated against THP-1 macrophages by determining half-maximal cytotoxic concentrations (CC_50_). The IC_50_ and CC_50_ values were determined by nonlinear regression analysis. Selected lead compounds underwent preliminary *in vitro* absorption, distribution, metabolism, and excretion (ADME) profiling. Synthesized analogs of both scaffolds were evaluated to investigate structure-activity relationships and opportunities for chemical optimization.

**Results:**

1,4-DAPP #9279817 and PTZ #9234527 were prioritized as lead compounds based on their combined antileishmanial activity and host-cell selectivity, exhibiting intracellular amastigote IC_50_ values of 3.50 and 3.12 μM and selectivity indices (SI) of 14.29 and 16.03, respectively. Singleton compounds exhibited variable parasite stage-specific activity, with host-cell cytotoxicity limiting the selectivity of several candidates. Both lead scaffolds retained activity across multiple *Leishmania* species, although compound susceptibility varied among species. Preliminary ADME profiling revealed distinct properties of the two leads, highlighting strengths and limitations for further optimization. Structural modifications of both scaffolds generated analogs with improved intracellular antileishmanial activity and selectivity.

**Conclusions:**

The 1,4-DAPP and PTZ scaffolds represent promising candidates for continued antileishmanial development. These findings support continued optimization of both scaffolds while emphasizing the importance of evaluating antileishmanial activity in disease-relevant parasite models.

## Background

Leishmaniasis is a neglected vector-borne tropical disease caused by obligate intracellular protozoan parasites of the genus *Leishmania* that are transmitted to mammals through the bite of infected female phlebotomine sand flies (1, 2). The disease is endemic in more than 90 countries and disproportionately affects populations in tropical and subtropical regions, particularly impoverished communities with limited access to healthcare (3). It is estimated that approximately 700,000 to 1 million new cases occur annually worldwide, representing a substantial global health burden. Leishmaniasis is classified by the World Health Organization as a neglected tropical disease (NTD), reflecting the limited therapeutic options and resources available for disease management (4).

More than 20 *Leishmania* species are capable of infecting humans, resulting in a broad spectrum of clinical manifestations that vary depending on infecting species and host immune status (5). Clinically, leishmaniasis presents in three primary forms, ranging from localized cutaneous infections to disseminated visceral disease, with clinical presentations influenced by the infecting species and host immune status. Cutaneous leishmaniasis (CL) is the most common form and is characterized by disfiguring skin lesions (6–8). Mucocutaneous leishmaniasis (MCL), primarily caused by parasites of the *Viannia* subgenera, can cause progressive destruction of mucosal tissue (9, 10). Visceral leishmaniasis (VL), the most severe form of disease, is characterized by systemic infections and enlargement of the internal organs such as the spleen and liver and is fatal in over 95% of symptomatic untreated cases (11–13). In addition to mortality, leishmaniasis is associated with substantial long-term morbidity, including permanent scarring, organ damage, and severe immunopathology.

*Leishmania* parasites possess a digenetic life cycle involving both the sand fly vector and mammalian hosts (14, 15). Within mammalian hosts, promastigotes are phagocytosed by macrophages and differentiate into intracellular amastigotes that replicate within the phagolysosomal compartments of macrophages (16, 17). This intracellular localization presents a significant challenge for therapeutic interventions, as candidate compounds must effectively penetrate the host cells and maintain activity within the acidic phagolysosomal environment.

Current treatment options for leishmaniasis rely on a limited number of therapeutic compounds, many of which possess significant clinical limitations (18). First-line antileishmanial treatments primarily include pentavalent antimonial such as sodium stibogluconate and meglumine antimonate, which have been in use for decades in endemic regions (19, 20). Although effective against the parasite, these treatments are often associated with severe toxicity, prolonged treatment regimens, and reduced efficacy due to the emergence of drug-resistant parasites (21, 22). Second-line antileishmanial treatments include compounds such as pentamidine, amphotericin B, paromomycin, and miltefosine (23, 24). While these compounds provide additional treatment options, their use remains limited by toxicity, high cost, route of administration, lengthy treatment regimens, and increasing reports of drug resistance (25). Despite decades of research and various candidates in clinical trials, there is no approved human vaccine available for leishmaniasis, further emphasizing the need for continued treatment development (26).

The development of therapeutics for neglected tropical diseases presents unique challenges due to limited financial investment, complex parasite biology, and the need for treatments that are safe, affordable, and deployable in resource-limited settings (27, 28). Increasing reports of antileishmanial drug resistance further complicate disease management and highlight the urgent need for compounds with novel mechanisms of action (29, 30). To address these challenges, standardized hit and lead criteria have been proposed for neglected tropical disease drug discovery programs, emphasizing parameters such as antiparasitic potency, selectivity, physicochemical properties, safety, oral bioavailability, and scalability of production (31). These target candidate profile (TCP) frameworks provide important guidelines for prioritizing compounds with translational potential and advancing early-stage hits through drug discovery pipelines. Within this framework, phenotypic high-throughput screening (HTS) approaches have emerged as valuable tools for identifying compounds with antiparasitic activity independent of a predefined molecular target. Unlike target-based approaches, phenotypic screening enables the identification of chemically diverse compounds acting through potentially novel or uncharacterized mechanisms (32).

Several large-scale phenotypic screening studies have successfully identified promising antileishmanial scaffolds, demonstrating the utility of HTS platforms for expanding the current therapeutic pipeline (33–35). However, many screening efforts focus on a single parasite species despite substantial biological and pharmacological differences among *Leishmania* species that may influence parasite susceptibility to therapeutic compounds (36, 37). Consequently, evaluating the candidate compounds across multiple clinically relevant species is important for determining the range and translational potential of antiparasitic activity (38).

In this study, we build upon previous high-throughput screening of the ChemBridge DIVERset-EXP chemical library to further characterize novel antileishmanial compounds. In addition to the previously identified 2,4-diaminoquinazoline (2,4-DAQ) scaffold (34), we characterize two additional lead scaffolds, 1,4-diaryl-pyrazolo-pyridinone (1,4-DAPP) and pyrazolo-[5,1-c][1,2,4]triazine (PTZ), as well as structurally distinct singleton compounds with antileishmanial activity. Candidate compounds were evaluated across parasite life stages and multiple *Leishmania* species. Representative 1,4-DAPP and PTZ compounds were further assessed through preliminary ADME profiling and structure-activity relationship (SAR) studies.

## Material and Methods

### Parasite and Cell Culture

*Leishmania tropica* (MHOM/AF/87/RUPERT), *L. infantum* (MHOM/TR/3/Adana7), transgenic *L. donovani* (MHOM/SD/62/1S-CL2D) constitutively expressing mCherry (39), and transgenic *L. major* (MHOM/JL/80/Friedlin) constitutively expressing mCherry (39) were maintained as promastigotes at 27°C in M199 medium (MP Biomedicals, Santa Ana, CA) supplemented with 10% FBS as previously described (40, 41). Axenic amastigotes were generated from log-stage *L. donovani* promastigotes in RPMI-1640 medium (Corning Life Sciences, Tewksbury, MA) supplemented with 10% fetal bovine serum and 1% penicillin-streptomycin (Corning) which was pH adjusted to 5.5 and maintained at 37°C as previously described (40). *Trypanosoma cruzi* (Dm28c) were maintained as epimastigotes at 27°C in Liver Infused Tryptose (LIT) media under standard culture conditions as previously described (42). Human THP-1 cells (ATCC TIB-202) were maintained at 37°C with 5% CO_2_ in RPMI-1640 medium (Corning) supplemented with 10% fetal bovine serum and 1% penicillin-streptomycin (Corning). THP-1 cells were differentiated into mature macrophages by incubation with 150 nM phorbol 12-myristate 13-acetate (PMA) for 24 h.

### Initial High-Throughput Screening

*Leishmania* promastigotes and axenic amastigotes were seeded at a density of 1 × 10^6^ parasites/mL in 96- and 384-well plates as previously described (34, 39). Compounds from the DIVERset library cassette #5 (ChemBridge, San Diego, CA, USA) were screened at a final concentration of 50 μM in appropriate media and DMSO controls and incubated for a minimum of 48 h. Viability of transgenic parasite lines constitutively expressing mCherry was determined via fluorescence (518 nm excitation/605 nm emission) using a FlexStation 3 Benchtop Multi-Mode Reader (Molecular Devices, San Jose, CA, USA). Viability of non-transgenic parasite lines was determined based on CellTiter-Blue (Promega, Madison, WI, USA) resazurin reduction (555 nm excitation/580 nm emission) following a 4 h incubation using a FlexStation 3 Benchtop Multi-Mode Reader (Molecular Devices). All assays were performed in at least two independent experiments with three technical replicates per condition.

### Secondary Screening and IC Determination

#### Promastigote Assays

*Leishmania* promastigotes were cultured under the conditions described above and seeded at a density of 1 × 10^6^ parasites/mL in 96-well plates. Parasites were incubated for 48 h in the presence of decreasing compound concentrations starting at 50 μM in appropriate media and DMSO controls. Viability of transgenic parasites was determined via fluorescence (518 nm excitation/605 nm emission) using a FlexStation 3 Benchtop Multi-Mode Reader (Molecular Devices). Viability of non-transgenic parasite lines was determined based on CellTiter-Blue (Promega) resazurin reduction (555 nm excitation/580 nm emission) following a 4 h incubation using a FlexStation 3 Benchtop Multi-Mode Reader (Molecular Devices). All assays were performed in at least three independent experiments with three technical replicates per condition.

#### Axenic Amastigote Assays

Axenic amastigotes were generated under the conditions described above. Axenic amastigotes were seeded at a density of 1 × 10^6^ parasites/mL in 96-well plates and incubated for 48 h in the presence of decreasing compound concentrations starting at 50 μM in appropriate media and DMSO. Viability of transgenic parasites was determined via fluorescence (518 nm excitation/605 nm emission) using a FlexStation 3 Benchtop Multi-Mode Reader (Molecular Devices). All assays were performed in at least three independent experiments with three technical replicates per condition.

#### Intracellular Amastigote Assays

*Leishmania* promastigotes and THP-1 macrophages were cultured under the conditions described above. Metacyclic promastigotes were isolated from stationary-phase parasite cultures using a Ficoll-400 density gradient as previously described (43). Differentiated THP-1 macrophages were infected with metacyclic parasites at parasite-to-macrophage a multiplicity of infection (MOI) of 10:1 for 6 h. Following infection, macrophages were washed with phosphate-buffered saline (PBS) to remove extracellular parasites and incubated overnight at 37°C with 5% CO_2_ in 96-well plates. Infected macrophages were incubated for 48 h in the presence of decreasing drug concentrations starting at 50 μM in appropriate media and DMSO controls. Viability of transgenic parasites was determined via fluorescence (518 nm excitation/605 nm emission) using a FlexStation 3 Benchtop Multi-Mode Reader (Molecular Devices). Following incubation, plates were washed with PBS and maintained at 27°C overnight to allow surviving intracellular amastigotes to transform back into promastigotes. Viability of non-transgenic parasite lines was determined based on CellTiter-Blue (Promega) resazurin reduction (555 nm excitation/580 nm emission) following a 4 h incubation using a FlexStation 3 Benchtop Multi-Mode Reader (Molecular Devices). All assays were performed in at least three independent experiments with three technical replicates per condition.

### Cytotoxicity Assay

#### THP-1 Macrophages

THP-1 cells were cultured and differentiated under the conditions described above. Briefly, THP-1 cells were differentiated into mature macrophages by incubation with 150 nM PMA for 24 h. Cells were washed with PBS to remove undifferentiated cells and incubated overnight at 37°C with 5% CO_2_ in 96-well plates. Macrophages were then incubated for 48 h in the presence of decreasing drug concentrations starting at 50 μM in appropriate media and DMSO controls. Viability of THP-1 macrophages was determined based on CellTiter-Blue (Promega) resazurin reduction (555 nm excitation/580 nm emission) following a 4 h incubation using a FlexStation 3 Benchtop Multi-Mode Reader (Molecular Devices). All assays were performed in at least three independent experiments with three technical replicates per condition.

#### Lead Compound ADME Profiling

Top candidates from the 1,4-DAPP (#9279817) and PTZ (#9234527) chemical scaffolds identified during secondary characterization were evaluated in standard *in vitro* ADME assays to characterize properties relevant to their continued development. Assays were performed at Q^2^ Solutions (Morrisville, NC, USA).

#### Structure-Activity Relationship (SAR)

In collaboration with the Warren Center for Drug Discovery at the University of Notre Dame, representative analogs of the 1,4-DAPP and PTZ scaffolds were generated through R-group modifications to evaluate preliminary structure-activity relationships and improve compound properties, including solubility and pharmacokinetic properties (44). Structural modifications were designed to assess the impact of substituent variation, heteroaryl incorporation, and modulation of lipophilicity on antiparasitic activity and selectivity, which is consistent with current SAR-driven drug discovery approaches focused on optimizing physicochemical and biological properties simultaneously. Structural modifications were introduced while maintaining the core scaffold architecture within each compound class. Analog design was performed iteratively, with compound optimization guided by biological activity and predicted physicochemical properties, a strategy commonly employed in modern hit-to-lead optimization workflows. Compounds were synthesized and characterized using standard medicinal chemistry approaches, including nuclear magnetic resonance (NMR) spectroscopy and liquid chromatography-mass spectrometry (LC-MS).

##### 1,4-DAPP Analogs

Representative 1,4-DAPP analogs were synthesized through a multi-component pyrazolo-pyridinone reaction using amino-pyrazoles, benzaldehydes, and Meldrum’s acid under microwave-assisted conditions in aqueous acetic acid. Subsequent functionalization of the pyrazole nitrogen was achieved using Buchwald-Hartwig coupling conditions with appropriate aryl and heteroaryl halides. Modifications to the 1,4-DAPP scaffold focused on improving aqueous solubility and reducing excessive aromatic character while maintaining antiparasitic potency, reflecting recent medicinal chemistry strategies aimed at balancing potency with compound-like properties and oral bioavailability. Compounds were purified by silica gel chromatography and obtained in low to moderate yields.

#### PTZ Analogs

Representative PTZ analogs were synthesized from commercially available amino-pyrazole intermediates through nitrite-mediated cyclization reactions under acidic conditions. Scaffold diversification was further achieved through Suzuki cross-coupling reactions using substituted brominated intermediates. Structural diversification of the PTZ scaffold was performed to evaluate the effects of aromatic substitution and polarity on biological activity and compound-like properties, as recent SAR studies have demonstrated that subtle structural modifications can significantly influence potency and selectivity. Final products were purified by silica gel chromatography and obtained in low to moderate yields. All synthesized analogs were subsequently evaluated in biological screening assays to establish preliminary SAR trends across both scaffold classes.

#### Statistical Analysis

The IC_50_ and CC_50_ values were calculated by nonlinear regression analysis using GraphPad Prism Version 11.0.1 for Mac OS. Selectivity index (SI) values were calculated as the ratio of CC_50_ to IC_50_ values.

## Results

### High-throughput screening identifies two enriched chemical scaffolds and structurally diverse singleton compounds

Previous high-throughput phenotypic screening of compounds within the ChemBridge DIVERset-EXP cassette #5 library identified 210 active compounds representing multiple chemical scaffolds (34). In the present study, we further characterized compounds belonging to two enriched scaffolds, 1,4-DAPP and PTZ, along with selected structurally diverse singleton compounds (Fig. 1). Of the 43 1,4-DAPP and 73 PTZ compounds represented within the library, 24 1,4-DAPP and 13 PTZ compounds demonstrated > 80% effectiveness in the preliminary screens. Further evaluation of these compounds identified three 1,4-DAPP and four PTZ compounds with IC_50_ values ≤ 10 μM against *Leishmania* amastigotes. Among the selected singleton compounds, two compounds demonstrated IC_50_ values ≤ 10 μM against *Leishmania* amastigotes. Cytotoxicity against THP-1 macrophages was assessed to evaluate parasite selectivity and prioritize compounds for further characterization.

### Life-stage activity and host-cell selectivity identify lead compounds within the 1,4-DAPP and PTZ scaffolds

Compounds within the 1,4-DAPP and PTZ scaffolds demonstrated antileishmanial activity that varied across *L. donovani* parasite life stages (Table 1). Among the 1,4-DAPP compounds, activity against promastigotes varied considerably, with IC_50_ values ranging from 6.64 to 48.44 μM. Increased activity against axenic amastigotes compared to promastigotes was observed for #9279817 and #9227165, while other compounds displayed limited activity against both parasite stages. Of the 1,4-DAPP compounds evaluated, #9279817 demonstrated the most favorable profile, with IC_50_ values of 6.64, 4.54, and 3.50 μM against promastigotes, axenic amastigotes, and intracellular amastigotes, respectively. With a THP-1 macrophage CC_50_ >50 μM, #9279817 exhibited corresponding selectivity indices of 7.53, 11.01, and 14.29.

Among the PTZ compounds, activity against promastigotes and axenic amastigotes was relatively consistent, with IC_50_ values ranging from 4.67 to 9.70 μM and 3.16 to 5.49 μM, respectively. Of the PTZ compounds evaluated, #9234527 retained low-micromolar activity across all parasite life stages, with IC_50_ values of 6.13, 5.49, and 3.12 μM against promastigotes, axenic amastigotes, and intracellular amastigotes, respectively. With a THP-1 macrophage CC_50_ >50 μM, #9234527 exhibited selectivity indices of 8.15, 9.11, and 16.03 across parasite life stages, respectively. Based on their combined antileishmanial activity across parasite life stages and favorable host-cell selectivity, 1,4-DAPP #9279817 and PTZ #9234527 were prioritized as lead compounds for further characterization and scaffold optimization.

#### Structurally diverse singleton compounds exhibit stage-dependent antileishmanial activity against L. donovani with variable host-cell selectivity

Structurally diverse singleton compounds demonstrated substantial variation in antileishmanial activity across *L. donovani* parasite life stages and host-cell selectivity (Table 1). Several singleton compounds exhibited potent activity against promastigotes, including #9292300, #9272957, #9217119, and #9283571, with IC_50_ values ranging from 0.77 to 2.82 μM. However, activity against promastigotes did not consistently predict activity against axenic amastigotes. Compound #9292300 retained low-micromolar activity against axenic amastigotes (IC_50_ = 4.16 μM), while #9213529 demonstrated increased activity against axenic amastigotes (IC_50_ = 7.58 μM) despite limited activity against promastigotes (IC_50_ >50 μM). In contrast, #9272957, #9217119, and #9283571 exhibited substantially reduced activity against axenic amastigotes relative to promastigotes, demonstrating pronounced life-stage differences among singleton compounds.

Despite strong antileishmanial activity observed among selected singleton compounds, host-cell cytotoxicity limited the selectivity of several candidates. For example, #9292300 exhibited a THP-1 macrophage CC_50_ of 4.27 μM, resulting in an axenic amastigote SI of 1.03 despite its low-micromolar antiparasitic activity. In contrast, #9213529 exhibited a THP-1 macrophage CC_50_ >50 μM, resulting in an axenic amastigote SI of 6.60. Collectively, these findings demonstrate that structurally diverse singleton compounds possess distinct parasite stage-specific activity and variable host-cell cytotoxicity, resulting in less favorable profiles relative to the prioritized 1,4-DAPP and PTZ lead compounds.

### Lead compounds display distinct preliminary ADME profiles

Following identification of 1,4-DAPP #9279817 and PTZ #9234527 as lead compounds, both compounds were evaluated using preliminary *in vitro* ADME assays to assess properties relevant to their continued development (Supplementary Table 1). The two compounds exhibited distinct profiles across hepatocyte clearance, cytochrome P450 inhibition, plasma protein binding, and aqueous solubility. PTZ #9234527 exhibited lower intrinsic clearance than 1,4-DAPP #9279817 across all species evaluated, with the greatest difference observed in human hepatocytes (6.92 mL/min/kg compared to 34.15 mL/min/kg, respectively). Both compounds exhibited inhibition of CYP2C9 at 10 μM (48.0% for 1,4-DAPP #9279817 and 52.4% for PTZ #9234527), while 1,4-DAPP #9279817 demonstrated minimal inhibition of CYP2D6 and CYP3A4 compared to PTZ #9234527 (4.0% and 0.0% compared with 34.14% and 7%, respectively). Both lead compounds exhibited extensive human plasma protein binding, with unbound fractions of 0.8% for #9279817 and 0.7% for #9234527. In contrast to its higher intrinsic clearance, #9279817 demonstrated greater aqueous solubility than #9234527 in both sodium phosphate buffer at pH 7.4 and HBSS. Collectively, these findings demonstrate distinct ADME profiles for the two lead compounds, supporting continued optimization of both chemical scaffolds.

### Structure-activity relationship studies identify analogs with enhanced antileishmanial activity

To evaluate the potential for further optimization of lead compounds within the 1,4-DAPP and PTZ scaffolds, synthesized analogs were evaluated for antileishmanial activity against *L. donovani* axenic and intracellular amastigotes and for cytotoxicity against THP-1 macrophages (Supplementary Tables 2 and 3). Structural substitutions within both scaffolds were designed to explore opportunities to improve metabolic stability, aqueous solubility, antileishmanial activity, and host-cell selectivity.

### Optimization of the 1,4-DAPP scaffold

All 28 synthesized 1,4-DAPP analogs exhibited THP-1 macrophage CC_50_ values > 50 μM, demonstrating limited host-cell toxicity at the maximum concentrations evaluated (Supplementary Table 2). Structural modifications based on the parent compound #9279817 resulted in variable activity against *L. donovani* amastigotes. Eighteen of the 28 analogs exhibited IC_50_ values < 10 μM against axenic amastigotes, with four exhibiting IC_50_ values < 5 μM. Similar efficacy was observed against intracellular amastigotes, with 18 analogs exhibiting IC_50_ values < 10 μM and 13 exhibiting IC_50_ values < 5 μM.

Several analogs demonstrated improved intracellular activity relative to the parent compound #9279817, which exhibited an intracellular amastigote IC_50_ of 3.50 μM and a selectivity index of 14.29. Notably, ND6008, ND6017, ND6034, and G18–1 exhibited intracellular IC_50_ values of 2.34, 2.36, 2.31, and 2.62 μM, respectively, with corresponding selectivity indices of 21.37, 21.19, 21.65, and 19.08. In contrast, other structural modifications resulted in substantial reductions in intracellular activity, demonstrating that antileishmanial potency was affected by substitutions within the 1,4-DAPP scaffold. Overall, these findings identify structural modifications capable of enhancing intracellular antileishmanial activity and selectivity while retaining a favorable host-cell cytotoxicity profile.

### Optimization of the PTZ scaffold

Structural modification based on the parent compound #9234527 resulted in substantial variation in antileishmanial activity and host-cell cytotoxicity. All 27 synthesized PTZ analogs exhibited IC_50_ values < 10 μM against *L. donovani* axenic amastigotes, including 11 analogs with sub-micromolar activity (Supplementary Table 3). Five analogs exhibited increased THP-1 macrophage cytotoxicity, with CC_50_ values ranging from 4.11 to 19.82 μM, and were excluded from subsequent intracellular amastigote evaluation. Of the remaining 22 analogs, 14 exhibited intracellular amastigote IC_50_ values < 10 μM, including nine with IC_50_ values < 5 μM.

Several analogs demonstrated improved intracellular activity relative to the parent compound #9234527, which exhibited an intracellular amastigote IC_50_ value of 3.12 μM and a selectivity index of 16.03. Notably, ND0012, ND0015, and ND0026 exhibited intracellular IC_50_ values of 2.22, 1.98, and 2.43 μM, respectively, with corresponding selectivity indices of 22.52, 25.25, and 20.58. However, enhanced axenic amastigote activity did not consistently translate to improved intracellular potency, as several analogs exhibiting sub-micromolar axenic activity demonstrated substantially reduced intracellular activity. Overall, structural modifications of the PTZ scaffold identified analogs with enhanced antileishmanial potency and selectivity, although increased host-cell cytotoxicity was observed with some modifications.

### Lead scaffolds and singleton compounds exhibit species-dependent antileishmanial activity

Selected 1,4-DAPP, PTZ, and singleton compounds were evaluated against promastigotes of multiple *Leishmania* species to determine whether antileishmanial activity was conserved across species (Table 2). Compounds within the 1,4-DAPP scaffold demonstrated variable activity against *L. major*, *L. infantum*, and *L. tropica*. Lead compound #9279817 retained activity across all three species evaluated, with IC_50_ values of 10.11, 14.74, and 4.78 μM, respectively. Other 1,4-DAPP compounds exhibited greater species-dependent differences, including #9227165, which demonstrated limited activity against *L. major* and *L. infantum* but increased efficacy against *L. tropica*. PTZ compounds demonstrated antileishmanial activity across the *Leishmania* species evaluated, although compound efficacy varied among both compounds and species. Lead compound #9234527 retained activity across all three species, while the other PTZ compounds exhibited distinct cross-species activity profiles. Differences in potency were the greatest against *L. infantum* promastigotes, where IC_50_ ranged from 14.79 to 23.50 μM. Structurally diverse singleton compounds demonstrated greater variability in cross-species activity. Several compounds retained activity against multiple species, including #9292300, whereas others exhibited pronounced differences in susceptibility between species. Collectively, these findings demonstrate that antileishmanial activity is not uniformly conserved across *Leishmania* species and support evaluation against multiple clinically relevant species during lead characterization.

#### 1,4-DAPP and PTZ compounds exhibit limited activity against T. cruzi epimastigotes

To evaluate whether the activity of the 1,4-DAPP and PTZ scaffolds exhibited broader antiparasitic activity, selected compounds were evaluated against *T. cruzi* epimastigotes (Supplementary Table 4). Among the 1,4-DAPP compounds, epimastigote IC_50_ values ranged from 12.31 to 50 μM, with lead compound #9279817 exhibiting the greatest activity (IC_50_ = 12.31; SI = 4.06) compared to the remaining 1,4-DAPP compounds. PTZ compounds similarly demonstrated reduced activity against *T. cruzi* epimastigotes relative to their activity against *Leishmania*, with lead compound #9234527 exhibiting limited activity (IC_50_ = 44.74 μM; SI = 1.12). Overall, the reduced activity observed in *T. cruzi* epimastigotes suggests that the activity of the 1,4-DAPP and PTZ compounds against *Leishmania* does not necessarily extend to other kinetoplastid parasites.

## Discussion

Further characterization of compounds identified through a previous high-throughput phenotypic screen identified the 1,4-DAPP and the PTZ scaffolds as promising starting points for antileishmanial drug development. Evaluation across *Leishmania* parasite life stages and characterization of host-cell cytotoxicity supported the prioritization of 1,4-DAPP #9279817 and PTZ #9234527 based on their combined antileishmanial activity and selectivity. Preliminary ADME profiling revealed distinct pharmacological properties and potential developmental limitations for each lead compound, while structure-activity relationship studies demonstrated that both scaffolds were able to be chemically optimized. Together these findings highlight the value of progressive characterization in prioritizing phenotypic screening hits for continued development.

Among the 1,4-DAPP compounds evaluated, #9279817 was selected as a lead compound as it demonstrated a favorable balance of antileishmanial efficacy and host-cell cytotoxicity, retaining low-micromolar activity across promastigotes, axenic amastigotes, and intracellular amastigotes. Its activity and selectivity against intracellular amastigotes are particularly encouraging, as this stage more closely represents the parasite within the mammalian host. Preliminary ADME profiling identified both favorable properties and opportunities for further optimization. Compared with the PTZ lead, #9279817 demonstrated greater aqueous solubility and minimal inhibition of CYP2D6 and CYP3A4, but exhibited greater intrinsic hepatic clearance in all models, suggesting that metabolic stability represents an important consideration for continued development. The 1,4-DAPP SAR studies further supported the potential of this scaffold, as structural modifications generated several analogs with improved intracellular activity and selectivity while maintaining limited THP-1 macrophage cytotoxicity. Additionally, reduction in antileishmanial activity was observed with specific substitutions, suggesting that antileishmanial potency is dependent on specific structural features within the scaffold. These findings demonstrate that the 1,4-DAPP scaffold can be optimized with chemical modifications and support continued optimization aimed at improving its pharmacological properties while maintaining intracellular activity and selectivity.

Among the PTZ compounds evaluated, #9234527 demonstrated promising activity against *L. donovani* but presented a distinct profile from the 1,4-DAPP lead. #9234527 retained low-micromolar activity across the parasite stages evaluated and demonstrated favorable selectivity against intracellular amastigotes. In contrast to 1,4-DAPP #9279817, PTZ #9234527 exhibited substantially lower intrinsic hepatic clearance across all models tested, suggesting greater metabolic stability *in vitro*. However, this advantage was accompanied by lower aqueous solubility and greater CYP2D6 inhibition, demonstrating that the two lead scaffolds present different opportunities for optimization. PTZ SAR studies further demonstrated the potential of the PTZ scaffold, with structural modifications producing substantial increases in axenic amastigote potency, including several analogs with sub-micromolar activity. Importantly, these improvements did not consistently translate to increased intracellular activity, and selected modifications resulted in increased host-cell cytotoxicity. Nevertheless, several PTZ analogs demonstrated improved intracellular potency and selectivity relative to the parent compound. These findings demonstrate that PTZ can be optimized, although further characterization should emphasize retention of intracellular activity and host-cell selectivity in addition to improved solubility.

Beyond the enriched scaffolds, the activity of structurally diverse singleton compounds demonstrates the chemical diversity captured through phenotypic screening while also illustrating limitations of prioritizing compounds based on potency alone. Several singleton compounds exhibited potent antileishmanial activity, but their profiles differed substantially across parasite stages and in host-cell cytotoxicity. For example, #9292300 retained low-micromolar activity against axenic amastigotes but exhibited cytotoxicity at a similar concentration, limiting its selectivity. In contrast, #9213529 demonstrated limited activity against promastigotes but increased activity against axenic amastigotes. These contrasting profiles demonstrate that a favorable IC_50_ in an initial screening assay does not necessarily identify the most promising compound for advancement. Instead, potency must be considered alongside host-cell selectivity and activity against parasite stages relevant to mammalian infection when prioritizing phenotypic screening hits.

The differences observed among the singleton compounds reflected a broader pattern of parasite stage-dependent susceptibility through this study. Activity against promastigotes did not predict activity against axenic amastigotes, and efficacy against axenic amastigotes did not necessarily predict improved activity against intracellular parasites. Although axenic amastigotes provide a useful model for compound screening as they mimic the acidic environment of the phagolysosome without requiring a host cell, they may not fully reproduce the environment encountered by amastigotes within infected macrophages. Differences in compound access to intracellular amastigotes, physiochemical conditions within the phagolysosomal compartment, and interactions with the host cell may contribute to differences between axenic and intracellular activity (45, 46). These findings support incorporation of intracellular amastigote assays during lead prioritization and optimization.

Differences in compound susceptibility were also observed across parasite species. Both lead compounds retained activity across multiple *Leishmania* species of clinical importance, although potency varied according to both species and individual compounds. Similar variation among other 1,4-DAPP, PTZ, and singleton compounds indicates that activity against *L. donovani* alone does not necessarily predict equivalent potency across other *Leishmania* species. While *L. donovani* represents an important species for antileishmanial screening due to its role in visceral leishmaniasis, evaluating across additional species provides a broader assessment of the potential spectrum of compound activity. This species-dependent activity extends beyond *Leishmania*, as activity against *T. cruzi* epimastigotes was generally reduced and the relative activity of the compounds was not conserved. For example, 1,4-DAPP #9279817 retained moderate activity against *T. cruzi*, while PTZ #9234527 exhibited substantially reduced activity. These findings suggest that activity within the identified scaffold is not universally conserved across kinetoplastids and emphasize the importance of evaluating compounds against relevant parasite species when defining their potential spectrum of activity.

Although 1,4-DAPP #9279817 and PTZ #9234527 demonstrated promising intracellular activity and selectivity, these properties alone are insufficient to establish their potential as therapeutic candidates. Current antileishmanial drug development requires compounds to combine efficacy against intracellular parasites with adequate selectivity, physicochemical properties, metabolic stability, exposure, and safety.

While the intracellular activity of the two leads supports their continued development, the ADME identified properties requiring further optimization, including intrinsic hepatic clearance for 1,4-DAPP #9279817 and aqueous solubility for PTZ #9234527. These findings support the continued advancement of both scaffolds through lead optimization aimed at improving their respective pharmacological properties.

Several limitations of the present study should guide further investigations. Cross-species activity was evaluated using promastigotes; therefore, these findings establish species-dependent differences in parasite susceptibility but do not necessarily predict activity against intracellular amastigotes of each species. Similarly, the evaluation of *T. cruzi* was limited to epimastigotes, and further studies using an infection model would be necessary to determine whether the observed activity extends to disease-relevant intracellular stages. Although structural modifications of the 1,4-DAPP and PTZ scaffolds were designed in part to address metabolic stability and solubility, the optimized analogs were primarily evaluated for antileishmanial activity and host-cell cytotoxicity. Additional ADME profiling of the most favorable analogs will be necessary to determine whether these modifications addressed the pharmacological limitations of the parent compounds. Subsequently, pharmacokinetic, tolerability, and *in vivo* efficacy studies will be required to determine whether favorable *in vitro* activity can translate to sufficient exposure and therapeutic efficacy. Furthermore, because these compounds were identified through phenotypic screening, their molecular targets and mechanism of action remain unknown. Mechanism characterization may provide insight into the observed parasite stage- and species-dependent differences, guide medicinal chemistry, and inform future resistance studies. Overall, the present study identifies 1,4-DAPP and PTZ as promising, chemically optimizable antileishmanial scaffolds and provides a foundation for their continued pharmacological and preclinical characterization.

## Supplementary Files

This is a list of supplementary files associated with this preprint. Click to download.

- GraphicalAbstract.docx
- SupplementaryTables.docx
- Tables.docx

## Figures and Tables

**Figure 1:**
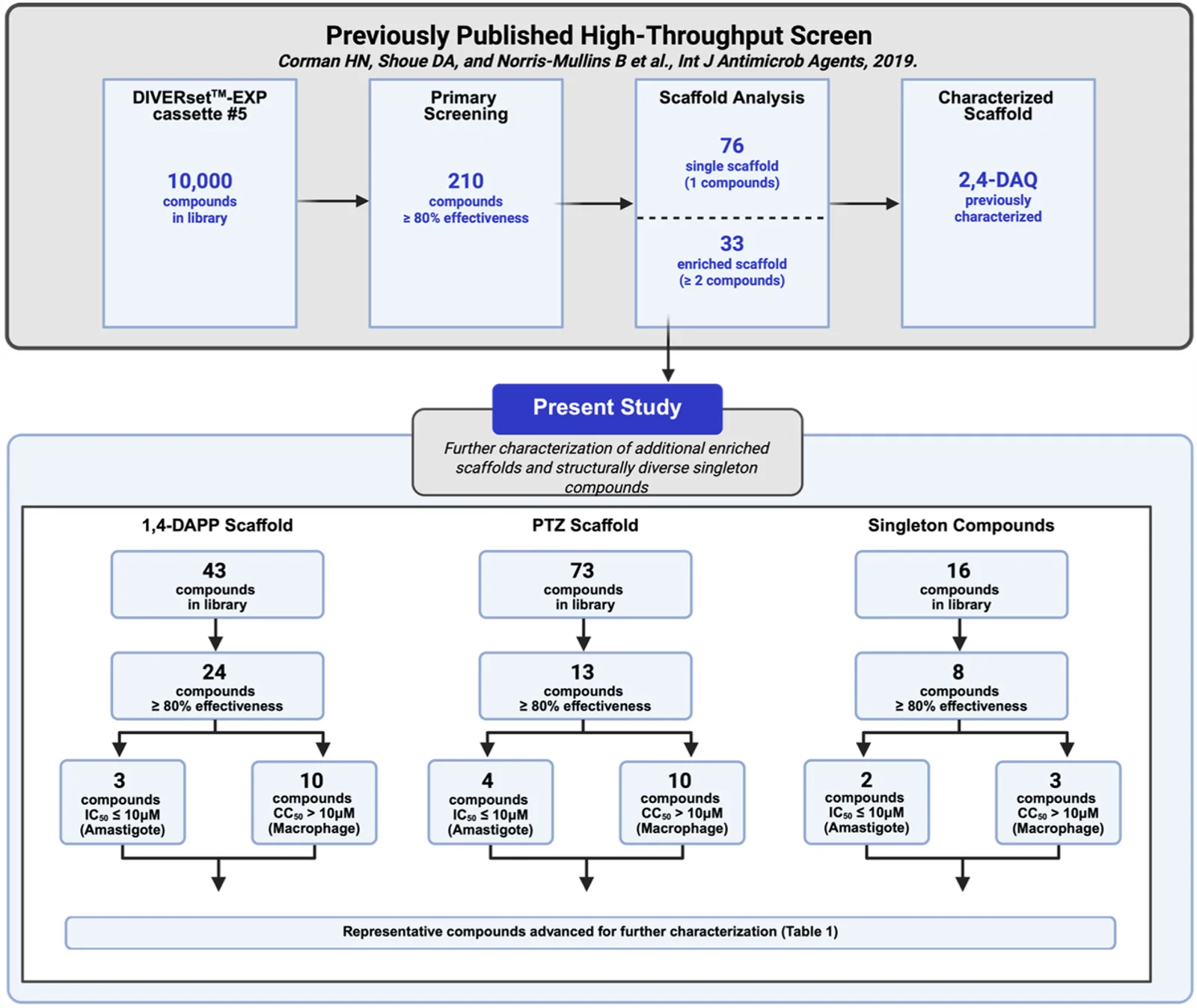
Identification and selection of candidate compounds from the DIVERset-EXP cassette #5 compound library for further characterization. The previously published high-throughput screen of 10,000 compounds identified 210 compounds with ≥80% effectiveness against *Leishmania donovani* and subsequently identified enriched chemical scaffolds within the library (34). In the present study, compounds belonging to the 1,4-diaryl-pyrazolo-pyridinone (1,4-DAPP) and the pyrazolo[5,1-c] [1,2,4]triazine (PTZ) scaffolds, as well as structurally diverse singleton compounds, were further evaluated. Compounds were prioritized based on antileishmanial activity (IC_50_ ≤10 μM) and macrophage cytotoxicity (CC_50_ >10 μM), representing compounds selected for further characterization.

## Data Availability

The raw data supporting the conclusions of this manuscript will be made available by the authors upon request.
